# Examining Compassionate Workplace Interventions for Employees Navigating Serious Illness, Caregiving, Death, or Bereavement: Protocol for a Scoping Review

**DOI:** 10.2196/82831

**Published:** 2026-05-01

**Authors:** Katie McGuinness, Karen Carr, Felicity Hasson, Esther Ruth Beck, Paul Slater

**Affiliations:** 1 Faculty of Life and Health Sciences, Ulster University, 2 - 24 York Street, Belfast, BT15 1AP, United Kingdom, 44 28 9536 7278

**Keywords:** public health, palliative care, workplace, compassion, bereavement

## Abstract

**Background:**

Globally, researchers have documented an emergence of public health approaches within palliative care, leading to the development of compassionate communities, a movement that seeks to build community capacity to support individuals at the end of life and those affected by death and loss. Compassionate communities emphasize a social model of care that complements formal palliative services by reducing suffering, enhancing well-being, and empowering community members through collective action. The Compassionate City Charter broadens this scope to include civic institutions, outlining recommendations for adoption within workplaces and educational settings. However, empirical evidence examining the adoption of compassionate practices within workplace contexts remains limited.

**Objective:**

This study aims to systematically map and explore international evidence regarding compassionate workplace interventions for employees in a workplace or organizational setting who are experiencing serious illness or are affected by caregiving, death, or bereavement.

**Methods:**

The Joanna Briggs Institute (JBI) framework will guide this review to systematically search and locate published articles reporting primary research. The following five stages will be conducted: (1) research question and objectives development; (2) search strategy development; (3) study and gray literature search and selection across 7 databases, including CINAHL Ultimate (EBSCO), Scopus, PsycINFO (OVID), Web of Science, ABI/INFORM (ProQuest), Business Source Ultimate (EBSCO), and MEDLINE (ProQuest); (4) data extraction and charting; and (5) data analysis and presentation. The JBI 3-step strategy will be applied to determine the search method and article selection. Articles will be assessed for inclusion by two reviewers independently and included if they describe a compassionate approach to supporting employees navigating serious illness, caregiving, grief, or bereavement. The review will use the PRISMA-ScR (Preferred Reporting Items for Systematic Reviews and Meta-Analyses Extension for Scoping Reviews) guidelines to present the results.

**Results:**

Data collection for this scoping review was conducted between July and August 2025. The extracted data are currently being organized and analyzed, with submission of the final manuscript anticipated in May 2026.

**Conclusions:**

This protocol outlines a scoping review that will represent the first comprehensive review to explore the international evidence on compassionate workplace interventions for employees navigating serious illness, caregiving, death, or bereavement.

## Introduction

### Background

Globally, researchers have documented an emergence of public health approaches within palliative care, leading to the development of compassionate communities [1]. Derived from the World Health Organization’s Ottawa Charter [2], this movement seeks to build community capacity to support individuals at the end of life and reduce inequities from those navigating chronic illness, dying, or bereavement.

The need for such an approach is further reinforced by Allan Kellehear’s 95% rule [3], which highlights that people who are seriously ill and dying only spend 5% of their final year of life in the direct care of health care services, relying instead on family, friends, and wider community networks for support. While no definitive definition exists, Vanderstichelen et al [4] describe compassionate communities as ones that invest in policy or structure to reduce suffering, promote well-being, provide community support, and empower members. The Compassionate City Charter broadens this scope to include civic institutions, outlining recommendations for adoption within workplaces and educational settings [5].

Employees may face serious illness or have to navigate caregiving, death, or bereavement at multiple points throughout their career [6]. These significant life events create ripple effects that impact communities, families, and everyone connected to them [7,8]. This ripple effect frequently extends into the workplace, leading to increased absenteeism and, in environments where compassion is lacking, fostering feelings of disconnection and isolation [9]. Compassionate communities advocate for shared responsibility for end-of-life care; however, the workplace, where employees spend a considerable proportion of their time, remains underexplored in this context [10].

There is a growing demand for workplaces to adopt more proactive approaches in supporting employees dealing with end-of-life challenges [11]. This includes developing strategies, policies, and practices that specifically address these often-overlooked life events, which are typically not covered by traditional workplace safety and health programs [4]. Organizational management scholars contend that compassion is transformative within organizations, not only promoting healing but also strengthening relationships among workforce members, building relational resources such as trust, and reinforcing shared values of interconnectedness [12].

Further evidence demonstrates that when compassion is present in the workplace setting, there are fewer errors, reduced absenteeism and presenteeism, lower staff turnover, and improved productivity [13]. Furthermore, fostering compassion can strengthen emotional and social connections among employees [14] and boost workplace morale [15]. Evidence also shows, when compassion is shown to others within the workforce, it reduces stress [16], lowers levels of mental ill health [17], and increases well-being [18].

Compassionate work environments are defined by practices that foster collective well-being by supporting individuals through four key dimensions: attending to others’ needs, understanding their experiences, empathizing with their challenges, and helping to alleviate their suffering [14]. However, Bergeron [19] identifies that most organizations and managers are not equipped to deal with grief and the associated discomfort, lacking both training and organizational support structures.

Despite these challenges, Dutton et al [20] and Kanov et al [21] argue that compassionate practices must be embedded within an organization’s architecture, creating contexts where compassion becomes a routine part of daily operation. These practices strengthen and sustain the health of individuals, teams, and organizations. Eldor [22] and Lilius et al [23] demonstrate how successful workplace compassion interventions can improve employee well-being and reduce negative outcomes like staff turnover. The positive effects of compassion in the workplace are supported by evidence that it can reduce absenteeism and increase morale [24].

### Study Objectives

This scoping review aims to systematically map and explore international evidence regarding compassionate workplace interventions for employees in the workplace or organizational setting experiencing serious illness or navigating caregiving, death, or bereavement, contributing to knowledge on compassionate communities and employee well-being, and supporting further development of compassionate workplaces.

### Developing the Research Question

The research team comprises experienced academic, palliative care, and health care professionals, who developed the research question following multiple rounds of discussion and consultations. The following research question will guide the review and was developed using the Joanna Briggs Institute’s (JBI) population, concept, and context (PCC) framework [25]: What evidence exists regarding compassionate interventions (C) for employees (P) facing serious illness or navigating caregiving, death, or bereavement in the workplace (C)?

The key questions of this review are as follows:

- What is the international evidence on compassionate workplace interventions that support employees navigating serious illness, caregiving responsibilities, death, or bereavement, and what are the contextual characteristics?
- What strategies, frameworks, and best practice guidelines inform the development of compassionate workplace interventions?
- What barriers have been identified during development and implementation?
- What are the identified literature gaps in compassionate workplaces?

## Methods

### Overview

The methodology for this scoping review has been developed in alignment with the JBI guidance for designing and conducting scoping reviews [26]. This protocol has been registered and is publicly available on the Open Science Framework (OSF) [27]. The final review will be reported and analyzed using the PRISMA-ScR (Preferred Reporting Items for Systematic Reviews and Meta-Analyses Extension for Scoping Reviews) guidelines [28]. Searches were conducted between the weeks of July 28 and August 11, 2025, and screening will begin thereafter.

### Search Strategy

The JBI 3-step search strategy will be used in this review [25].

First, a preliminary search of MEDLINE (ProQuest) and CINAHL Ultimate (EBSCO) was conducted on May 12, 2025, with the assistance of an expert subject librarian to inform the development of a comprehensive search strategy. Initial searches used keywords, MeSH (Medical Subject Headings) terms and subject headings where appropriate. The preliminary search served as a pilot and involved analysis of retrieved articles to refine and analyze the search strategy.

In the second step, a comprehensive search was undertaken using keywords and subject headings (where appropriate) across all databases between July 28 and August 11, 2025, with title and abstract screening beginning on August 12, 2025, and full-text screening commencing on August 19, 2025. Following team discussions, the inclusion of mixed disciplinary databases has been identified as appropriate to best address the research question and objectives. The review includes the following databases: CINAHL Ultimate (EBSCO), Scopus, PsycINFO (OVID), Web of Science, ABI/INFORM (ProQuest), Business Source Ultimate (EBSCO), and MEDLINE (ProQuest).

These databases will provide a valuable range of perspectives and allow for in-depth searching through their advanced functionalities. The search strategy was adapted for each included database using a Boolean operator combination (“OR” “AND”), along with truncations (*) to combine search terms (see Multimedia Appendix 1 for complete PsycINFO search strategy).

In the third step, all included publications underwent backward and forward citation tracking to identify additional relevant studies not captured in the database searches. A gray literature search was also undertaken to identify unpublished literature that may be relevant to this review. These searches included PhD theses and dissertations, a gray literature database (TRIP), and Google Scholar to identify any relevant third sector and government organization web pages or documents. As per JBI guidance, the first 5 to 10 pages of Google’s search results were screened for relevant content [25].

The review follows the PRISMA-ScR checklist [28] to ensure transparency and rigor throughout the process (Checklist 1).

### Inclusion Criteria

The inclusion criteria for this review follow the JBI PCC framework and support the identification, collection, and extraction of data relevant to the research question and objectives [26].

#### Population

This review considered studies focusing on employees navigating serious illness, caregiving responsibilities, death, or bereavement within workplace settings.

#### Concept

This review considered studies reporting on the design, development, implementation, and evaluation of compassionate workplace strategies. These may include compassionate practices, information dissemination, development of educational materials, training initiatives, awareness initiatives, provision of support, and policy development and implementation.

#### Context

The target context for this review was studies conducted in diverse organizational settings across different geographical locations.

#### Limitations

Searches were limited to English-language publications, with studies published between 1998 and 2025 included. This language restriction is due to resource constraints and may result in the exclusion of relevant evidence from non–English-speaking contexts, representing a limitation of the review. The search start date of 1998 was selected as it marks the emergence of compassionate workplace concepts, first documented by Frost [29].

### Types of Evidence to Be Included

After searches are conducted, all identified articles will be collated and uploaded into Covidence, a web-based collaboration software program for systematic and literature reviews, where duplicates will be removed [30].

A pilot screening process will be conducted before the full screening. Two independent reviewers (KM and KC) will screen a random sample of titles and abstracts to assess the inclusion/exclusion criteria and ensure interrater reliability. Any disagreements will be discussed, and the inclusion criteria will be redefined. Where necessary, study authors will be contacted to obtain additional information required for inclusion decisions. Any disagreements between reviewers will be resolved through discussion to reach consensus; if necessary, a third reviewer will be used (FH or ERB) to ensure final inclusion decisions. The results of the search and study inclusion process will be reported in a PRISMA-ScR flow diagram [28].

### Data Extraction

A draft extraction form adopted from JBI guidance [31] will be piloted for appropriateness and comprehensiveness against a sample of 3 to 5 full-text articles by two researchers (KM and KC), with findings discussed with the research team. Upon agreement, data will be extracted from articles included in the scoping review by one reviewer (KM).

To enhance analytical coherence across a wide range of compassionate workplace interventions, compassion will be defined during data extraction as workplace strategies that aim to recognize, respond to, or reduce employee suffering related to serious illness, caregiving, death, or bereavement. Key information regarding the study and compassionate practices will be extracted, including intervention details, implementation strategies, outcomes, barriers and facilitators identified, and key findings, to address the research questions and objectives.

### Critical Appraisal of Available Literature

A formal critical appraisal of individual studies will not be undertaken, as this is not typically required for scoping reviews, which aim to map the breadth and nature of available evidence rather than assess methodological quality.

### Data Analysis and Presentation of Results

A qualitative content analysis will be conducted using a combined inductive-deductive approach, integrating both conventional and directed approaches [32]. Inductive category formation will enable categories to be derived directly from the retrieved articles, while deductive categories will be based on the predetermined PCC framework [31]. This dual approach allows for comprehensive categorization that captures both emerging themes from the literature and systematic organization according to the review’s objective and question. A narrative summary will accompany the tabulated results, describing how findings relate to the review’s objective and question. Reporting will align with PRISMA-ScR guidelines [28].

### Patient and Public Involvement

The target population or the public were not involved in the planning or conducting of this research. While consistent with scoping review methodology, this may limit the applied relevance of the findings. Findings will be shared with the Research advisory group supporting the wider PhD study to support interpretation and inform potential actions.

### Ethical Considerations

Ethics approval will not be sought for this study, as only readily available material will be used within the review.

## Results

Data collection for this scoping review was completed between July and August 2025. Screening and data extraction have been conducted and are currently being organized and analyzed. Submission of the completed scoping review is anticipated in May 2026.

## Discussion

### Anticipated Findings

This scoping review aims to systematically map and explore the existing international evidence on compassionate workplace interventions designed to support employees experiencing serious illness, caregiving responsibilities, death, or bereavement within organizational settings. The review seeks to identify the types of compassionate workplace interventions implemented across diverse workplace contexts, the strategies or frameworks used to guide their development, and the barriers and facilitators influencing their implementation.

To our knowledge, there is a limited body of synthesized evidence examining how compassionate approaches are developed, embedded, and operationalized within organizations internationally. While previous studies have explored compassion in the workplace [9,33], particularly focusing on bereavement support in workplace contexts [34,35], few have systematically examined compassionate interventions for employees experiencing serious illness, caregiving responsibilities, death, or bereavement across diverse organizational and international contexts. The absence of synthesized evidence may contribute to inconsistency and variability in workplace practices and support mechanisms for such employees.

A strength of this review lies in its ability to identify current practices, highlight gaps in the literature, and provide an evidence base where one does not currently exist. It is anticipated that this review will identify areas where organizational policies and practices may be undeveloped or inconsistently applied, highlighting opportunities to strengthen compassionate workplace knowledge and practice. Furthermore, by mapping the available evidence, the findings may inform the development of structured compassionate workplace strategies and interventions to support employee well-being at a challenging time in their lives.

Ultimately, this review will contribute to advancing awareness of the compassionate communities concept and encourage its application within workplace and organizational settings. The findings of this review are expected to inform future research and support the development of compassionate workplace practices for employees navigating serious illness, caregiving responsibilities, death, or bereavement.

### Limitations of the Study

Although the scoping review will be conducted in accordance with the JBI methodology for scoping reviews, some limitations may remain. The inclusion of studies from a range of organizational settings, geographical locations, and methodological designs may introduce heterogeneity in the evidence base, thus potentially limiting the ability to compare and synthesize findings.

The search strategy, although designed to be comprehensive and reproducible using established methodological guidance, is restricted to 7 databases and English-language publications, which may result in the exclusion of relevant published evidence in other languages or those not indexed within the selected databases. Additional efforts will be made to identify gray literature, though it is possible some relevant organizational practices or interventions may not be formally documented or publicly available.

Finally, consistent with the exploratory nature of scoping reviews, findings will reflect the evidence available at the time of search and may not capture subsequently published studies.

### Conclusion

This scoping review will systematically map and explore international evidence regarding compassionate workplace interventions, identifying current practices and highlighting knowledge gaps in the literature. The review will contribute to advancing understanding of compassionate workplaces within the broader compassionate communities concept and support further development of compassionate workplace practices in response to employees navigating serious illness, caregiving responsibilities, death, or bereavement.

Findings will be disseminated via peer-reviewed publications, conference presentations, and engagement with relevant stakeholders. This review represents the first stage of a wider program of research, with subsequent work expected to build on the findings to further explore compassionate workplace practices.

## Supplementary material

10.2196/82831 Multimedia Appendix 1 Database search strategy for PsycINFO (OVID).

10.2196/82831 Checklist 1 PRISMA-ScR checklist.

## Abbreviations

JBI: Joanna Briggs Institute
MeSH: Medical Subject Headings
OSF: Open Science Framework
PCC: population, concept, and context
PRISMA-ScR: Preferred Reporting Items for Systematic Reviews and Meta-Analyses Extension for Scoping Reviews

## References

[1] Abel J (2018). Compassionate communities and end-of-life care. Clin Med (Lond).

[2] (1987). Ottawa Charter for Health Promotion. World Health Organization.

[3] Kellehear A (2020). Compassionate cities: global significance and meaning for palliative care. Prog Palliat Care.

[4] Vanderstichelen S, De Moortel D, Nielsen K (2024). Developing and evaluating compassionate workplace programs to promote health and wellbeing around serious illness, dying and loss in the workplace (EU-CoWork): a transdisciplinary, cross-national research project. Palliat Care Soc Pract.

[5] Wegleitner K, Heimerl K, Kellehear A (2016). Compassionate Communities: Case Studies from Britain and Europe.

[6] Flux L, Hassett A, Callanan M (2020). Grieving in the workplace: how do grieving employees perceive their experience of workplace support from management?. Pol Pract Health Saf.

[7] Brossoit RM, Crain TL, Leslie JJ, Hammer LB, Truxillo DM, Bodner TE (2019). The effects of sleep on workplace cognitive failure and safety. J Occup Health Psychol.

[8] Benevene P, Buonomo I, West M (2022). Editorial: compassion and compassionate leadership in the workplace. Front Psychol.

[9] Lilius JM, Worline MC, Maitlis S, Kanov J, Dutton JE, Frost P (2008). The contours and consequences of compassion at work. J Organ Behavior.

[10] Mills J, Abel J, Kellehear A (2024). The role and contribution of compassionate communities. Lancet.

[11] Bakelants H, Van Droogenbroeck F, De Donder L (2026). Developing a compassionate university: insights from a longitudinal process evaluation. Death Stud.

[12] Dutton JE, Frost PJ, Worline MC, Lilius JM, Kanov JM (2002). Leading in times of trauma. Harv Bus Rev.

[13] (2025). Compassionate workplaces training programme. Marie Curie.

[14] Atkins PWB, Parker SK (2012). Understanding individual compassion in organizations: the role of appraisals and psychological flexibility. Acad Manage Rev.

[15] Frost PJ, Dutton JE, Worline MC, Wilson A, Fineman S (2000). Emotion in Organizations.

[16] Pace TWW, Negi LT, Adame DD (2009). Effect of compassion meditation on neuroendocrine, innate immune and behavioral responses to psychosocial stress. Psychoneuroendocrinology.

[17] Wilson AC, Mackintosh K, Power K, Chan SWY (2019). Effectiveness of self-compassion related therapies: a systematic review and meta-analysis. Mindfulness (N Y).

[18] Zessin U, Dickhäuser O, Garbade S (2015). The relationship between self-compassion and well-being: a meta-analysis. Appl Psychol Health Well Being.

[19] Bergeron DM (2023). Time heals all wounds? HRM and bereavement in the workplace. Hum Resour Manag Rev.

[20] Dutton JE, Workman KM, Hardin AE (2014). Compassion at work. Annu Rev Organ Psychol Organ Behav.

[21] Kanov JM, Maitlis S, Worline MC, Dutton JE, Frost PJ, Lilius JM (2004). Compassion in organizational life. Am Behav Sci.

[22] Eldor L (2018). Public service sector: the compassionate workplace—the effect of compassion and stress on employee engagement, burnout, and performance. J Public Adm Res Theory.

[23] Lilius JM, Worline MC, Dutton JE, Kanov JM, Maitlis S (2011). Understanding compassion capability. Hum Relations.

[24] Simpson AV, Pina e Cunha M, Rego A (2014). Compassion in the context of capitalistic organizations: evidence from the 2011 Brisbane Floods. J Bus Ethics.

[25] Peters MDJ, Marnie C, Tricco AC (2020). Updated methodological guidance for the conduct of scoping reviews. JBI Evid Synth.

[26] Peters MD, Godfrey C, McInerney P, Munn Z, Tricco AC, Khalil H, Aromataris E, Lockwood C, Porritt K, Pilla B, Jordan Z (2024). JBI Manual for Evidence Synthesis JBI.

[27] McGuinness K, Slater P, Carr K, Beck E, Hasson F (2025). Compassionate workplace interventions for employees navigating serious illness, caregiving, death, or bereavement: a scoping review protocol. Open Science Framework.

[28] Tricco AC, Lillie E, Zarin W (2018). PRISMA Extension for Scoping Reviews (PRISMA-ScR): checklist and explanation. Ann Intern Med.

[29] Frost PJ (1999). Why compassion counts!. J Manag Inq.

[30] (2025). Covidence.

[31] Pollock D, Peters MDJ, Khalil H (2023). Recommendations for the extraction, analysis, and presentation of results in scoping reviews. JBI Evid Synth.

[32] Elo S, Kyngäs H (2008). The qualitative content analysis process. J Adv Nurs.

[33] Bakelants H, Van Droogenbroeck F, Chambaere K (2024). A compassionate university for serious illness, death, and bereavement: qualitative study of student and staff experiences and support needs. Death Stud.

[34] Gilbert S, Mullen J, Kelloway EK, Dimoff J, Teed M, McPhee T (2021). The C.A.R.E. model of employee bereavement support. J Occup Health Psychol.

[35] Flux L, Hassett A, Callanan M (2019). How do employers respond to employees who return to the workplace after experiencing the death of a loved one? A review of the literature. Pol Pract Health Saf.

