# Supplementary material for: Examining Compassionate Workplace Interventions for Employees Navigating Serious Illness, Caregiving, Death, or Bereavement: Protocol for a Scoping Review
Source: JMIR Res Protoc. 2026 May 1;15:e82831. doi: 10.2196/82831 (PMC13134823; doi:10.2196/82831)
Supplement: Multimedia Appendix 1 [file resprot-v15-e82831-s001.docx]

| Step | Search Terms |
| --- | --- |
| 1 | Exp Chronic illness/ or exp Acute illness/ or exp Critical Illness/ or exp Chronic Mental Illness/ |
| 2 | Caregivers/ |
| 3 | “death and dying”/ |
| 4 | bereavement/ or grief/ |
| 5 | palliative care/ |
| 6 | 1 or 2 or 3 or 4 or 5 |
| 7 | (illness* or care giv* or death or bereave* or palliative or end of life or dying).mp. (mp=title, abstract, heading word, table of contents, key concepts, original title, tests & measures, mesh word) |
| 8 | 6 or 7 |
| 9 | (compassion* adj3 (workplace or work place* or institution* or organization* or organizations* or employe* or work environment* or place* of work)).mp. (mp=title, abstract, heading word, table of concepts, original title, tests & measures, mesh word) |
| 10 | 8 and 9 |
| 11 | Limit 10 to (English language and yr=”1998 -Current”) |
